# Hand hygiene adherence at entrances and exits of healthcare facilities in two rural districts of Uganda

**DOI:** 10.1017/ash.2022.144

**Published:** 2022-05-16

## Abstract

**Background:** During the COVID-19 pandemic, the World Health Organization (WHO) has recommended hand hygiene (HH) stations (ie, with soap and water for handwashing or alcohol-based hand rub or ABHR) at entrances and exits of every public or private commercial building, including healthcare facilities (HCFs). **Methods:** Enumerators observed the HH materials present at the entrances and exits of 37 public HCFs in the Moroto and Kotido districts and patient and visitor use of those HH materials. When handwashing stations were nonfunctional or out of water, no HH observations were made. **Results:** Of the 37 HCF entrances and exits assessed, 4 (11%) met the recommended guidance for HH materials: 3 (8%) had water and soap, and 1 (3%) had ABHR and water and soap. In other HCFs, 12 (32%) had no HH station present, 13 (35%) handwashing stations had no water, and 8 (22%) had water but not soap. Of 180 persons observed, 52 (29%) attempted HH and only 10 (6%) used appropriate HH technologies (4 with ABHR and 6 with water and soap). Of 52 people who attempted HH, 42 (81%) used only water without soap. All HH observed occurred when entering facilities; no HH occurred when exiting (0 of 68). Of those 52 who performed HH, 48 (92%) performed HH for the recommended time of >20 seconds. However, only 9 (5%) of 180 adhered to suggested HH technologies and length of time (used water and soap scrubbing for ≥20 seconds or used ABHR). **Conclusions:** We detected poor HH practice by patrons at entrances and exits of HCFs, which may be due to lack of appropriate HH materials, particularly lack of soap. Optimal strategies for adherence to WHO-recommended HH practices at entrances and exits of public and private commercial buildings, including HCFs, should be explored.

**Funding:** None

**Disclosures:** None

